# Estimating individual optimal sleep duration and potential sleep debt

**DOI:** 10.1038/srep35812

**Published:** 2016-10-24

**Authors:** Shingo Kitamura, Yasuko Katayose, Kyoko Nakazaki, Yuki Motomura, Kentaro Oba, Ruri Katsunuma, Yuri Terasawa, Minori Enomoto, Yoshiya Moriguchi, Akiko Hida, Kazuo Mishima

**Affiliations:** 1 Department of Psychophysiology, National Institute of Mental Health, National Center of Neurology and Psychiatry, 4-1-1, Ogawa-Higashi, Kodaira, Tokyo, 187-8553, Japan

## Abstract

In this study, we hypothesized that dynamics of sleep time obtained over consecutive days of extended sleep in a laboratory reflect an individual’s optimal sleep duration (OSD) and that the difference between OSD and habitual sleep duration (HSD) at home represents potential sleep debt (PSD). We found that OSD varies among individuals and PSD showed stronger correlation with subjective/objective sleepiness than actual sleep time, interacting with individual’s vulnerability of sleep loss. Furthermore, only 1 h of PSD takes four days to recover to their optimal level. Recovery from PSD was also associated with the improvement in glycometabolism, thyrotropic activity and hypothalamic-pituitary-adrenocortical axis. Additionally, the increase (rebound) in total sleep time from HSD at the first extended sleep would be a simple indicator of PSD. These findings confirmed self-evaluating the degree of sleep debt at home as a useful clinical marker. To establish appropriate sleep habits, it is necessary to evaluate OSD, vulnerability to sleep loss, and sleep homeostasis characteristics on an individual basis.

Sleep is an essential behavior that takes up around one third of a human lifetime, but securing sufficient sleep time is difficult in modern society. The US Centers for Disease Control and Prevention reports that 30% of workers in the United States sleep less than 6 h/day^1^. Sleep loss (or sleep debt) causes sleepiness and a decline in performance^2^,^3^ and also impairs many other psychological and physical functions such as memory, learning^4^, metabolism^5^, and immunity^6^,^7^. However, little is known about the amount of sleep need to maintain normal functioning of the mind and body.

Epidemiological studies have expressed the relationship between sleep time and various health risks (e.g., cardiovascular disease^8^,^9^,^10^, obesity and metabolism^11^,^12^, depression^13^, and mortality rate^14^,^15^,^16^,^17^) as a U-shaped curve with 7–8 h at the trough. Similarly, a study investigating the relationship between sleep time and psychomotor vigilance performance reported a sleep need of 8.16 h for preventing cumulative neurobehavioral deficits^18^. When Klerman and Dijk^19^ gave young and elderly subjects the opportunity to sleep 16 h/day, sleep time showed exponential decay in both groups and asymptotic values were obtained at 8.9 h and 7.4 h, respectively. In addition, significant individual differences in habitual sleep duration (HSD) are well recognized, with a normal distribution of 5–10 h^20^,^21^,^22^. There have also been reports that individuals who exhibit long or short HSD over an extended period exhibit a change in nocturnal intervals of high melatonin levels^23^ and in the effects of genetic variations^24^. Therefore, the evidence to date makes clear that deciding on a standard of amount of sleep need across the board is difficult and that individual differences must be considered.

Sleep is believed to be controlled and maintained by the joint activity of two separate mechanisms, homeostasis and circadian rhythm^25^,^26^. With sleep loss (i.e., an extended awake time), the homeostasis carries over the residual sleep pressure and attempts to resolve the carryover at the next opportunity for sleep. We hypothesize that optimal sleep duration (OSD) is reflected in sleep time when sleep homeostasis resolves sleep debt through the compensatory mechanism of extended sleep. We also hypothesize that OSD differs among individuals and that the difference between HSD and OSD represents the state of “potential” sleep debt (PSD), which the individuals do not clearly recognize. Therefore, in this study, we conducted a sleep extension experiment that provided healthy participants with the opportunity to sleep 12 h each night over 9 consecutive days in an attempt to estimate OSD and quantify PSD on an individual basis.

## Results

### Habitual sleep duration

To estimate OSD and PSD, we recruited 15 healthy young men (mean age, 23.1 ± 2.1 years; range, 20–26 years). They recorded their daily HSD at home using an actigraph and sleep diary for approximately 2 weeks. Their mean morningness-eveningness score (MEQ score) derived from the Japanese version of Morningness-Eveningness Questionnaire^27^,^28^ was 46.3 ± 7.2, and mean sleep-corrected midpoint of sleep in free days (MSFsc) derived from Japanese version of Munich ChronoType Questionnaire^29^,^30^ was 5.0 ± 1.3 h.

They then started a 14-day sleep protocol in a sleep laboratory experiment consisting of time in bed (TIB) set at 8 h for an adaption night (AN) and a baseline night (BL), then 9 consecutive days of extended sleep sessions (sessions E1–E9) with TIB set at 12 h followed by 1 night of total sleep deprivation (SD) and 1 night of 12-h recovery sleep (RS) (see Supplementary Fig. S1). Among all participants, the mean HSD at home was 7.37 ± 0.27 h (range, 5.82–8.89 h). For each individual, HSD also significantly varied by day, with a mean range in HSD of 6.36 ± 0.82 h (range, 2.03–14.42 h; see Supplementary Fig. S2).

The mean total sleep times (TSTs) at AN and BL were 7.35 ± 0.08 h and 7.39 ± 0.09 h, respectively, which were not significantly different from HSD (F (1.1,15.398) = 0.013, p = 0.928 with Greenhouse-Geisser correction for sphericity).

### Estimating optimal sleep duration and potential sleep debt

**Figure 1:**
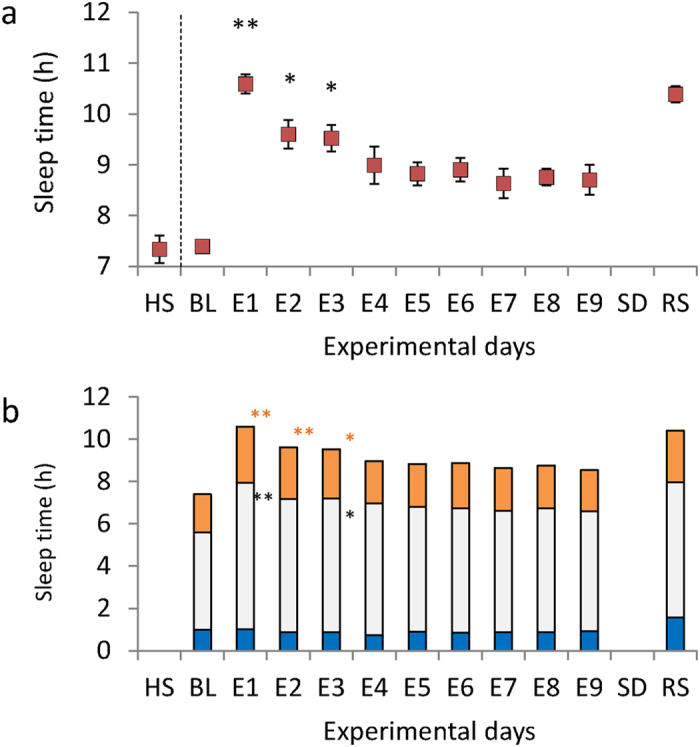
Total sleep time (TST) and sleep structure during the sleep protocol. (**a**) TST as determined by polysomnography (PSG) on each experimental night. BL: baseline night; E1–E9: extended sleep sessions on days 1–9; SD: sleep deprivation night; RS: recovery sleep night. Habitual sleep (HS) is the total sleep time calculated from at-home actigraphy. Data are expressed as means with error bars representing the standard error of the mean. **p < 0.01, *p < 0.05. Mixed model analysis of variance for repeated measures showed significant differences in TST within extended sleep sessions (F (8,109.107) = 10.590, p < 0.001; HS, BL, and RS not included in the analysis). Multiple comparisons showed TST was significantly longer at E1, E2, and E3 compared to E9. (**b**) Sleep structure determined by PSG on each experimental night. Blue filled bar: slow wave sleep; Grey filled bar: sleep stages 1 + 2 (st1 + 2); Yellow filled bar: REM sleep (stREM). **p < 0.01, *p < 0.05. During extended sleep sessions, the total amount of sleep showed significant differences in st1 + 2 and stREM, but not sleep stages 3 + 4 (st3 + 4) (st1 + 2, F(8,110.012) = 5.973, p < 0.001; stREM, F(8,110.164) = 8.003, p < 0.001; st3 + 4: F(8,109.970) = 1.342, p = 0.230). Multiple comparisons showed E1 and E3 had longer st1 + 2 and stREM than E9, and E2 showed longer stREM than E9.

The mean TST showed a distinct successive change over E1–E9. The mean TST at E1 was 10.59 ± 0.19 h and showed a large increase (rebound) from HSD (mean, 3.22 h), but decreased through extended sleep sessions over the next 8 days (F (8,109.107) = 10.590, p < 0.001; Fig. 1a). There were no significant differences in TST between each of E4–E8 and E9. Over the extended sleep sessions, the total durations of stage 3 + 4 (st3 + 4; known as slow wave sleep) showed no significant changes (F (8,109.970) = 1.342, p = 0.230). In contrast, shallow non-rapid eye movement (NREM) sleep (stages 1 + 2; st1 + 2) and REM sleep (stREM) were significantly higher at E1, E2, and E3 compared to E9 (st1 + 2: F (8,110.012) = 5.973, p < 0.001; stREM: F(8,110.164) = 8.003, p < 0.001; Fig. 1b).

From the exponential decay curve fitting for the decrease in TST over the 9 days of extended sleep sessions, we were able to obtain an asymptote (the estimation of OSD) from individual TSTs (R^2^ = 0.244–0.946; see Supplementary Fig. S3) for 13 participants. TSTs for 2 of the participants (S05 and S11) did not converge for curve fitting. These results indicate that sleep debt did not exist for these 2 participants, and therefore instead of an asymptote, we treated the mean TST value over the extended sleep sessions as the estimate of OSD.

**Figure 2:**
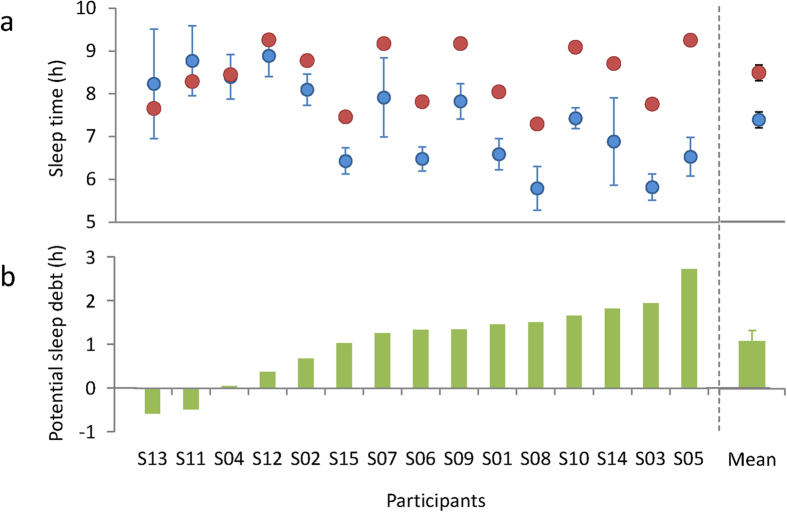
Habitual sleep duration (HSD), optimal sleep duration (OSD), and potential sleep debt (PSD) for each individual. (**a**) Mean and individual’s OSD and HSD. Individual data are presented in ascending order of PSD. Red filled circles: OSD, Blue filled circles: HSD. (**b**) Mean and individual’s PSD (HSD subtracted from OSD). Mean HSD was significantly shorter than mean OSD by approximately 1 h (t(14) = 4.547, p < 0.001).

The mean estimated OSD was 8.41 ± 0.18 h (range, 7.29–9.26 h). The range of OSD was smaller than the range of HSD (Fig. 2a). There was a significant correlation between estimated OSD and HSD (r = 0.514, p = 0.050; see Supplementary Fig. S4), suggesting that OSD is a determinant of sleep habits, even in actual daily life where society’s schedule plays a role. However, HSD was significantly shorter than OSD (t(14) = 4.547, p < 0.001; Fig. 2a), and the result of the trial calculation of mean PSD (i.e., the difference between OSD and HSD) for the 15 participants was 1.04 ± 0.24 h (range, −0.58 to 2.73 h; 95% confidence interval: 0.57–1.58 h; Fig. 2b). We thus observed a relationship where PSD increased as HSD became shorter (r = −0.756, p = 0.001; see Supplementary Fig. S4). On the other hand, there was no significant correlation between PSD and OSD (r = 0.172, p = 0.539; see Supplementary Fig. S4).

It is shown in most studies that circadian typology (morningness-eveningness preferences or chronotype) is associated with the homeostatic sleep regulation^31^. In this study, however, there are no significant correlations, neither between MSFsc and PSD (r = −0.220, p = 0.431) or OST (r = −0.072, p = 0.799), nor between MEQ score and PSD (r = 0.230, p = 0.409) or OST (r = −0.067, p = 0.812).

**Figure 3:**
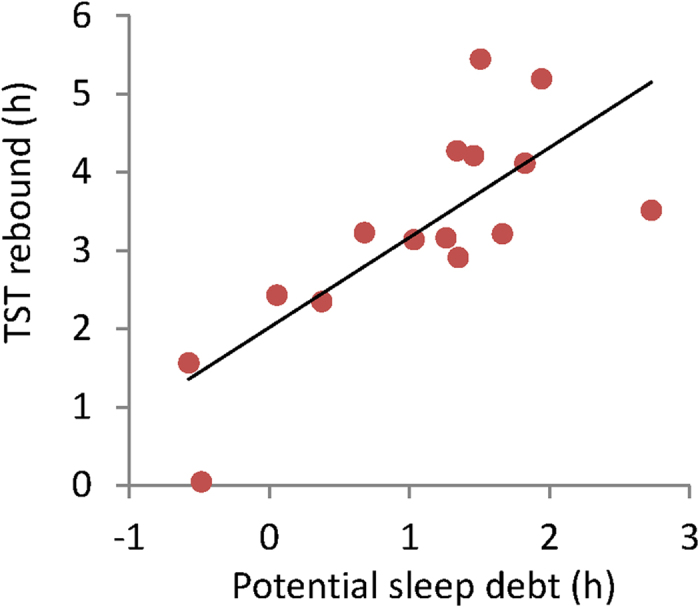
Association between potential sleep debt and total sleep time (TST) rebound. TST rebound is the difference between TST at E1 and habitual sleep duration. The greater the potential sleep debt, the significantly greater the TST rebound (r = 0.769, p = 0.001).

There was a significant correlation between the TST rebound at E1and PSD (r = 0.769, p = 0.001; Fig. 3). In other words, compensatory adjustment was observed in response to sleep loss in each individual. In addition, because there was no significant correlation between OSD and TST at E1 (r = 0.228, p = 0.414), we believe TST rebound is determined by both E1 and HSD.

### Association of potential sleep debt with objective sleepiness

In regard to subjective sleepiness (determined using a visual analogue scale) and objective sleepiness (determined with the Maintenance Wakefulness Test), recovery was achieved in E1–E3 and reached a level equal to that of E9 (Visual analogue scale: F(9,126) = 7.346, p < 0.001) or that of E8 (Maintenance Wakefulness Test: F(8,112) = 3.968, p < 0.001) (see Supplementary Fig. S5).

HSD showed no correlation with visual analogue scale or Maintenance Wakefulness Test at BL (Visual analogue scale: r = 0.077, p = 0.785; Maintenance Wakefulness Test: r = 119, p = 0.673). On the other hand, there was a significant trend of correlation between PSD and Maintenance Wakefulness Test (r = −0.486, p = 0.066) but no correlation between PSD and visual analogue scale (r = 0.052, p = 0.854; Table 1, see Supplementary Fig. S6). These results suggest that it is difficult to estimate daytime sleepiness from HSD alone, and that even PSD, which consists of a relationship between the individual’s OSD and HSD, has only a modest correlation with Maintenance Wakefulness Test. However, there are individual differences in vulnerability to sleep debt^32^,^33^, and it is thus possible that the correlation between PSD and objective sleepiness will be diminished. Accordingly, we a multiple regression analysis using the general mean slow wave activity (SWA) power^34^,^35^ as a covariate, obtained from the first NREM sleep cycle in RS, which is believed to reflect sleep homeostasis. As a result, PSD showed a significant negative correlation with Maintenance Wakefulness Test score (β = −0.544, p = 0.029; Table 1).

### Association of potential sleep debt with neuroendocrine functions

Effects of sleep extension for 9 days on neuroendocrine functions were summarized on Table 2. In regard to glycometabolic function, there was a significant decrease in plasma glucose concentration (t = 2.153, p = 0.05) and trends for increase in HOMA-β (t = −2.023, p = 0.061) at E9 compared to BL. In regard to thyroid function, there was a significant increase in serum TSH (t = −6.102, p < 0.001) and T4 (t = −3.054, p = 0.009) concentration at E9 compared to BL. In regard to hypothalamus-pituitary-adrenocortical (HPA) axis function, there was a significant decrease in plasma ACTH (t = 2.601, p = 0.021) and cortisol (t = 3.091, p = 0.008) concentration at E9 compared to BL.

## Discussion

This study is the first to estimate, on an individual basis, PSD from differences in HSD and estimated OSD, which was obtained by repeated, extended sleep sessions that offered participants the opportunity for 12 h sleeps over 9 consecutive days.

The mean at-home sleep time (i.e., HSD) was 7.3 h in the participants, whose mean age was 23.4 years. According to a national survey^36^ conducted in 2011, the mean sleep time for Japanese men in the same age cohort (20–24 years) is similar, at 7.56 h. Sleep time in epidemiological studies is a survey item that is close to TIB. Also, given the tendency for individuals to overestimate sleep time, it seems that the participants in the present study had average sleep habits typical for their age. In fact, the participants had no awareness of any problems with their sleep habits such as sleepiness or perceived sleep loss. However, even in this group of healthy individuals who subjectively reported having enough sleep, we observed a TST rebound of more than 3 h in average at E1. Thus, they were experiencing possible sleep loss without being aware of it. Thereafter, TST decreased, reached standard OSD levels at E4, and leveled off. Subjective sleepiness showed a similar decreasing pattern as sleep time approached OSD, but objective sleepiness recovered at E1. Such a fast disappearance of objective sleepiness is consistent with published findings that a decline in cognitive behavioral performance due to a rather strong sleep debt recovers after only 1 night of recovery sleep^37^. The period required for sleep time to reach the OSD (the period for fulfilling sleep) may therefore be related to the time course of recovery for biological functions other than sleepiness and cognitive behavioral performance. The role shallow sleep plays in the time course for biological recovery may be reflected in the finding that compensatory sleep extension was seen in st1 + 2 and stREM but not deep sleep (st3 + 4).

This finding relates, for example, to a decline in metabolism or the immune function^5^,^38^,^39^, large-scale changes in the blood transcriptome^40^,^41^, and other impairments of biological functions as a result of the preferential appearance of deep sleep, which in turn can cause partial sleep deprivation such as declines in st1 + 2 and stREM.

The mean OSD for the present participants was 8.41 ± 0.18 h. This value is basically consistent with the asymptotic level for TST in young people reported in previous sleep extension research^19^,^42^ as well as the threshold reported to cause a decrease in cognitive behavioral performance in dose-dependency studies of partial sleep deprivation^18^. It seems reasonable, therefore, to estimate that the mean sleep time needed for young adults is around 8.5 h. Indeed, many epidemiological studies suggest that sleeping less than 6 h or more than 9 h is related to an increased mortality, heart disease, obesity, and mood disorders^8^,^9^,^10^,^11^,^12^,^13^,^14^,^15^,^16^,^17^.

The present study also found individual differences in OSD, distributed over a range of about 2 h. These results were obtained in an isolated experimental room that strictly controlled for any factors such as activities and food that might influence sleep pressure, so it is possible that the individual differences seen in OSD are governed by endogenous factors and are, in part, genetically determined. A twin study involving about 4500 twin pairs reported that the contribution of genetic factors to sleep time is around 30% and that this finding was stable over 15 years of follow up^43^. Also, point mutations in *DEC2*, a clockwork gene for biological time, have been found among humans who are short sleepers, and mice with *DEC2* mutations have shown the same decrease in sleep time^24^.

In this study, OSD and HSD exhibit a trend where sleep habits followed the sleep need of each individual, but most participants (13/15) had HSD lower than OSD. The mean PSD was approximately 1 h but with a range of 3 h among the 15 participants. PSD did not correlate with OSD but showed a high correlation with HSD (r = 0.76). This suggests that short sleep times in daily life, more than individual differences in sleep need, contribute to the accumulation of PSD. On the other hand, PSD, not HSD, showed a trend of correlation with Maintenance Wakefulness Test score at BL. Additionally, the relation between PSD and Maintenance Wakefulness Test score became more robust after adjusting for individual differences in sleep homeostasis (i.e., SWA in the first NREM sleep cycle of RS). These findings clearly indicate that a tailor-made approach to each individual’s necessary sleep times, rather than an absolute amount for sleep, is an effective way to approach risk assessment and sleep debt repayment.

We also observed remarkable effects of recovery from sleep debt on various neuroendocrine functions, in that extended sleep induced significant decrease in plasma glucose concentration and trends for increase in HOMA-β, which is an index of insulin secretory function derived from fasting plasma glucose and insulin concentrations^44^. Previous cross-sectional studies have shown that decreased HOMA-β correlated risks for the incidence of type 2 diabetes^45^. TSH and T4 were increased after sleep extension in comparison to the baseline. Normally TSH was inhibited by sleep, especially by slow wave sleep (SWS), therefore acute total sleep deprivation leads to the increase of TSH^20^. While partial sleep deprivations were association with the inhibition of TSH and inductions of T3 and T4 in short-term^39^,^46^ and inhibitions of TSH and T4 in long-term^47^. Our findings were in line with those of the latter study. In previous study with rodents, sleep deprivation would decrease the TRH mRNA^48^. Thus, sleep extension may results in the increase of the production and/or secretion of TSH in pituitary, and subsequent inductions of peripheral T4. Furthermore, sleep extension resulted in the decrease in the ACTH and cortisol secretion level. Previous studies have reported that short sleep duration is associated with higher cortisol level^39^,^49^. Our finding suggests the risk that even mild to moderate (~1 h) PSD could also induce the hyperactivity of HPA system.

In reality, however, there are large costs associated with assessing individual OSD or PSD using the present method. In clinical settings, TST rebound that showed a strong correlation with PSD may be a useful and simple indicator for the presence of sleep debt. TST rebound is analogous to catch-up sleep on the weekends from sleep debt that occurs as a result of social obligations such as going to work or school. The increase in TST on a weekend or holiday indicates that PSD has a strong relationship with sleepiness rather than with HSD as the previous studies implicated^50^,^51^. Suppressing this increase as much as possible may be one approach to establishing sleep habits suited to each individual.

The present findings revealed that healthy young adults who were not aware of sleep problems were experiencing approximately 1 h of PSD every day, and that opportunities for sufficient sleep up to 9 days are needed to eliminate the sleep debt. The recovery from PSD also correlated with normalization of daytime sleepiness, sleep structure and some neuroendocrine functions including the glycometabolic and stress-related hormones. Recovery sleep periods required for these physiological function might vary widely. From a different angle, it could be proposed that modest but long-term sleep deprivation (potential sleep debt) may be one of the causes of growing number of patients with obesity, diabetes mellitus and mood disorders. In addition, we suggest that HSD by itself cannot determine the occurrence of sleepiness due to sleep debt because it is also determined by PSD, which is dependent on an individual’s sleep need or OSD and sleep homeostasis. This suggests that to establish appropriate sleep habits, it is necessary to consider OSD and sleep homeostasis, which differ according to the individual’s vulnerability to sleep loss. We hope the present findings can contribute to future studies on the biological basis of sleep debt. Further research is also required on the genetic factors contributing to sleep debt.

## Methods

### Participants

Sixteen healthy young men (mean age, 23.4 ± 2.4 [SD] years; range, 20–26 years) volunteered for this study. The health of all participants was confirmed by questionnaire, interview with a doctor, biochemical blood tests, brain structure imaging and functional magnetic resonance imaging (fMRI), and overnight clinical polysomnography. All participants were non-smokers, were not currently on any medication, and had no physical, psychological, or sleep disorders. In addition, none had engaged in shift work that included night shifts or had traveled with more than 6 h of jet lag in the 3 months prior to the experiment. Of these 16 participants, 1 withdrew from the experiment, but not due to any physical or psychological problems at the time of dropping out. Thus, data from 15 participants (mean age, 23.3 ± 2.1 years; range, 20–26 years) were subjected to analysis.

### Procedures

All participants completed a 14-day sleep laboratory experiment in the isolation laboratory of the National Center of Neurology and Psychiatry, after recording data at home for an average of about 2 weeks. The details of the study and explanation of its aims were explained to the participants in writing and their written informed consent was obtained. This study was conducted following approval by the Medical Research Center Ethics Committee, National Neurology and Psychiatry, Japan. The present study was conducted according to the principles of the Declaration of Helsinki.

Prior to the laboratory experiment, daily HSD was recorded by a wrist actigraph (MicroMini Motionlogger Actigraph, Ambulatory Monitoring, Inc.) worn on the participants’ wrist of the non-dominant hand, set at 1-min epochs and in zero crossing mode. The accuracy of the records was confirmed by descriptions and times self-reported in an online sleep diary the participants completed daily before and after sleep. When naps during the day were recorded in the sleep diary and by the actigraph, the equivalent time slot was considered a daytime sleep interval. Sadeh’s Algorithm^52^ was applied to each daytime and nighttime sleep interval against the amount in the activity records, using Action W-2 software (version 2.6.9905, Ambulatory Monitoring, Inc.). The TST obtained from both day and night readings were combined to represent the individual’s HSD. Participants were also administered the Japanese version of Morningness-Eveningness Questionnaire (MEQ)^27^,^28^ and the Japanese version of Munich ChronoType Questionnaire (MCTQ)^29^,^30^ to evaluate their circadian typology.

After the at-home recordings were completed, all participants were introduced to the 14-day sleep protocol in the laboratory setting. The schedule in each room was established relative to the participant’s at-home records. The average bedtime calculated for each participant was set as 0 (24) h. The participant entered the room at 17:00 on day 1, ate, took a bath, and went to bed, with the lights turned off at 24:00.

For nights 1 and 2 in the laboratory—AN and BL, respectively—TIB was set to 8 h in accordance with each participant’s habitual bedtime. Data for AN were excluded from the analysis. For nights 3–11, the extended sleep times in sessions E1–E9 were set to 12 h, with bedtime set 2 h earlier. After waking on E9, the participants had 39 h of SD. After SD, they had 12 h of RS (started 1 h after the participant’s habitual bedtime). After waking from RS, measurement devices were removed and the experiment was concluded.

During the sleep protocol, the participants lived in the laboratory without any contact with the outside world through cell phones or the Internet. During the planned wake periods, they were asked to stay awake in a room with interior luminance (approx. 100 lx) and at the time to sleep, the lights were turned off (approx. 0 lx) and the participants were asked to sleep in the bed. Experimenters continuously observed the participants and if unintended naps occurred, the experimenter verbally woke the participants. During the wake periods, the participants were allowed to move around the laboratory, write, read, listen to music, watch videos, play video games, and speak to the experimenters, but physical exercise and unplanned meals and drinks were prohibited. The experimentation room temperature and humidity were set at 25 ± 0.5 °C and 50 ± 5% relative humidity.

All periods of sleep in the laboratory were recorded within a soundproof sleep room by all-night PSG, using a digital PSG data recording system (EEG-1200, Nihon Kohden, Ltd., Tokyo Japan). Both left and right electrooculograms (EOG), submental myograms (EMG), electrocardiograms (ECG), and electroencephalograms (EEG) from 9 areas (F_3_-A_2_, F_4_-A_1_, C_3_-A_2_, C_4_-A_1_, O_1_-A_2_, O_2_-A_1_, F_z_-A_1_, C_z_-A_1_, P_z_-A_1_) were digitized. All signals were passed through a low-pass filter with a cutoff frequency of 30 Hz and a high-pass filter with a time constant of 0.3 s (sampling rate: 200 Hz). Two technicians trained in reading PSG recordings and blinded to the experimental conditions determined sleep stages from 30-s epochs according to standard criteria^53^. TST, sleep efficiency (%TST relative to TIB), wake time, REM sleep, st1, st2, and slow wave sleep (st3 + 4) were determined from PSG and st1 and st2 were summed. The PSG records for 2 of the participants (E4 of S07, E6 of S11) were not used due to technical problems.

For frequency analysis, the power value was calculated in 2-Hz bins in the 0–20 Hz range from EEG data in NREM sleep derived from Fz on the recovery night by fast Fourier transform (Hanning window, 2.5-s epoch). NREM sleep cycles were determined in accordance with Feinberg and Floyd’s^54^ criteria. SWA was defined in the range of 0.5–4.75 Hz. The SWA power of each NREM sleep cycle obtained was normalized by the average power value of all NREM sleep cycles, and from these data, the SWA power of the first NREM sleep cycle was used as the indicator of sleep homeostasis for each participant.

Every day during the sleep protocol, excluding day 1 of TSD, the Maintenance Wakefulness Test was conducted around 19:00 (5 h before habitual bedtime). On day 2 of TSD, the Maintenance Wakefulness Test was conducted at around 23:00. Both times the test was conducted in the soundproof sleep room, following the recommendation of the American Academy of Sleep Medicine^55^. On the bed, the participants leaned back on a traditional legless chair with a head support and maintained a semi-recumbent posture, with their eyes open, and were requested to try not to fall asleep. The test started from the time the lights were turned off (from the time the lights were dropped to low environmental illumination, 0.10–0.13 lx) and ended when the participant fell asleep or after 40 min from lights out. The EEG derivation for the Maintenance Wakefulness Test and montage were the same as for PSG, and C_3_–A_2_/C_4_–A_1_ was used to determine the moment of falling asleep. Sleep latency (from lights out to falling asleep) was recorded as the Maintenance Wakefulness Test score. If the test finished at 40 min without the participant falling sleep, the Maintenance Wakefulness Test score was recorded as 40 min.

During the wake periods throughout the sleep protocol, the participants were given the visual analogue scale to complete every 0.5–2 h and subjectively evaluated their degree of sleepiness, mood, and appetite. The mean visual analogue scale score obtained during the wake period each day were taken as the representative value of that day.

A blood sample was drawn every day after awakening (around 11:00). The levels of glycometabolism (insulin and glucose), thyroid hormone (thyroid-stimulating hormone (TSH) triiodothyronine (T3) and free thyroxin (T4)) and stress hormone (cortisol and adrenocorticotropic hormone (ACTH) were determined by a commercial biochemistry laboratory (SRL, Inc., Tokyo, Japan). The homeostasis model assessment of insulin resistance index (HOMA-IR) and HOMA of percent beta-cell function (HOMA-β) were calculated from fasting plasma insulin and glucose levels as insulin in μIU/mL * glucose in mg/dL/405, and (360 * insulin in μIU/mL)/(glucose in mg/dL *− 63)%, respectively^39^.

fMRI and T1-weighted MRI images were acquired between around 13:00 and 15:00 (9–11 h before habitual bedtime) on day 2 (BL), day 7 (day before E5), day 12 (day 1 of TSD), and day 13 (day 2 of TSD) and 18:00–20:00 (4–6 h before habitual bedtime) on day 1 (AN), day 12 (day 1 of TSD), and day 13 (day 2 of TSD). The analysis of brain imaging data will be reported elsewhere.

### Statistical analysis

We utilized a mixed model analysis of variance for repeated measures to analyze daily variations in TST, each sleep stage (st1 + 2, st3 + 4, and stREM), and subjective and objective sleepiness measured by the visual analogue scale and Maintenance Wakefulness Test, respectively. For fixed effects, we used TST, extended sleep sessions (E1–E9) for the model of each sleep stage, and the experimental days from BL to E9 for subjective and objective sleepiness. To estimate OSD, the following exponential decay curve was fitted to the TST obtained from PSG for all extended sleep sessions (E1–E9):


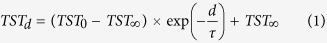


Here, TST_d_ denotes TST for each extended sleep session, TST_0_ is TST at the start of the extension (i.e., the intercept at the Y-axis), d is days, τ is the time constant of the decay curve, and TST_∞_ is the lower bound of the estimated TST.

For exponential decay curve fitting, we used the constrained nonlinear regression module of IBM SPSS Statistics Version 21.0. The initial value of TST_0_ was set to TIB (12); the initial value of τ was set to the constant estimated from linear interpolation by linear regression between E1 and E5, obtained from the estimate of TST of all 15 participants (i.e., the estimated number of days until the difference between E1 and E5 showed a decrease of 37% (2.52)); and the initial value of TST_∞_ was set to the lowest value (8.63) of the mean TST of all 15 participants. We imposed the constraint that TST_∞_ ≥ 0. The fit did not converge for 2 of the 15 participants (S05 and S11). We interpreted this to indicate that no rebound due to sleep deprivation occurred, and thus considered the mean TSTs from the extended sleep sessions to be the individual OSDs for these participants. Because PSG records were not usable for 2 participants (E4 for S07 and E6 for S11) due to technical problems, curve fitting was conducted for 8 nights of their PSG records. Also, there was extremely low sleep efficiency (<50%) for only E9 in 1 participant (S14) and thus the data from that night were excluded from the curve fitting and PSG records from the remaining 8 nights were used.

OSD and HSD were compared using a paired t-test. Pearson’s correlation analysis was used to examine the relationship between parameters.

The relationships of PSD with subjective and objective sleepiness were examined by hierarchical regression analysis (crude: PSD only input; adjusted: PSD and sleep homeostatic characteristics input). Forced input was used, with the sleepiness index as the dependent variable and PSD and sleep homeostatic characteristics as the explanatory variables.

The levels of glycometabolism, thyroid hormones and stress hormones before (BL) and after (E9) sleep extension were compared using a paired t-test.

All results are expressed as means  ±  SEM. All statistical analysis was conducted using IBM SPSS Statistics Version 21.0 (IBM Corporation). Statistical significance was set to 5%.

## Additional Information

**How to cite this article**: Kitamura, S. *et al*. Estimating individual optimal sleep duration and potential sleep debt. *Sci. Rep.* **6**, 35812; doi: 10.1038/srep35812 (2016).

## Supplementary Material

Supplementary Information

## Figures and Tables

**Table 1:** Correlations between potential sleep debt and sleepiness parameters.

Variable	Visual analogue scale		Maintenance Wakefulness Test		
	β	P	β	P	
*CRUDE*					
Potential sleep debt	0.052	0.854	−0.486	0.066	
*ADJUSTED (Forced entered)*					
Potential sleep debt	0.067	0.820	−0.544	0.029*	
SWA in RS 1^st^ cycle	0.118	0.0691	−0.447	0.064	

SWA: slow wave activity; RS: Recovery sleep.

**Table 2:** Effects of sleep extension for 9 days on neuroendocrine functions.

Variable	BL	E9	*t*	*P*
	Mean ± SEM	Mean ± SEM		
Insulin (μIU/mL)	5.2 ± 0.7	5.6 ± 0.5	−0.978	0.345
Glucose (mg/dL)	92.1 ± 1.4	90.4 ± 1.1	2.153	0.050*
HOMA-IR	1.2 ± 0.2	1.3 ± 0.1	−0.693	0.499
HOMA-β	62.3 ± 5.4	73.0 ± 5.9	−2.023	0.061+
Thyroid-stimulating hormone (TSH) (μIU/ml)	1.3 ± 0.1	2.0 ± 0.1	−6.102	< 0.001*
Triiodothyronine (T3) (pg/ml)	2.9 ± 0.1	3.0 ± 0.1	−0.985	0.343
Thyroxin (T4) (pg/ml)	1.2 ± 0.0	1.3 ± 0.0	−3.054	0.009*
Adrenocorticotropic hormone (ACTH) (pg/ml)	13.4 ± 1.1	11.4 ± 0.7	2.601	0.021*
Cortisol (μg/dl)	22.8 ± 3.3	18.8 ± 2.3	3.091	0.008*

BL: Baseline; E9: 9^th^ day of Extended sleep; HOMA-IR: homeostasis model assessment of insulin resistance index; HOMA-β: HOMA of percent beta-cell function. *p < 0.05, +p < 0.10.
