# Supplementary material for: Estimating individual optimal sleep duration and potential sleep debt
Source: Sci Rep. 2016 Oct 24;6:35812. doi: 10.1038/srep35812 (PMC5075948; doi:10.1038/srep35812)
Supplement: Supplementary Information [file srep35812-s1.pdf]

Supplementary Information

Estimating individual optimal sleep duration and potential sleep debt

Shingo Kitamura[1], Yasuko Katayose[1], Kyoko Nakazaki[1], Yuki Motomura[1], Kentaro Oba[1], Ruri Katsunuma[1], Yuri Terasawa[1], Minori Enomoto[1], Yoshiya Moriguchi[1], Akiko Hida[1], and Kazuo Mishima[1]

1 Department of Psychophysiology, National Institute of Mental Health, National Center of Neurology and Psychiatry, 4-1-1, Ogawa-Higashi, Kodaira, Tokyo, 187-8553, Japan

Address correspondence to: Kazuo Mishima, M.D., Ph.D., Department of Psychophysiology, National Institute of Mental Health, National Center of Neurology and Psychiatry, 4-1-1 Ogawa-Higashi, Kodaira, Tokyo 187-8553, Japan; E-mail: mishima@ncnp.go.jp

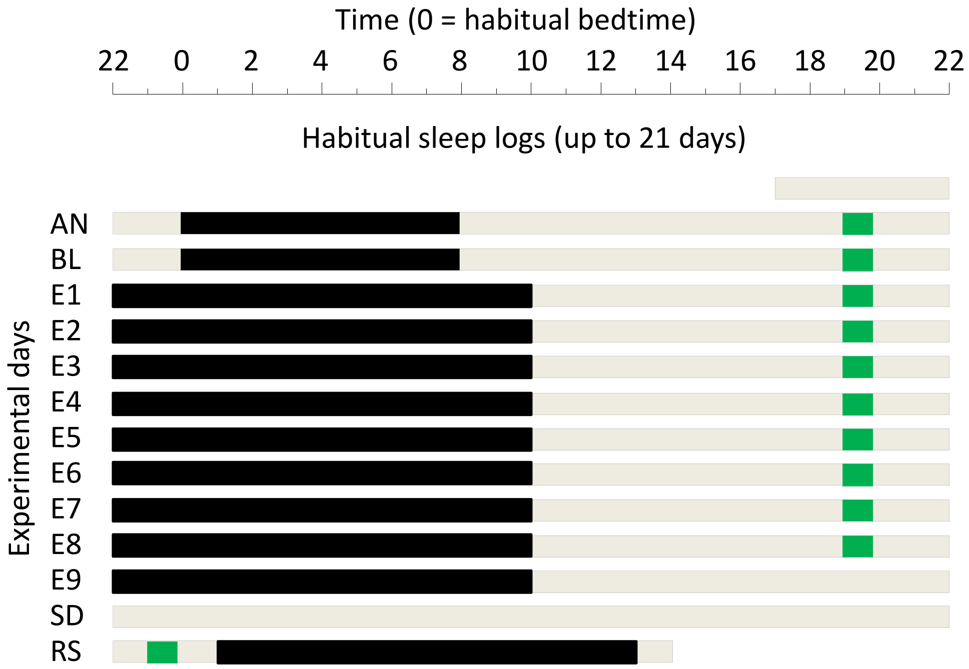

Figure S1 Experimental protocol

■ Sleep period (≒0 lux) □ Wake period (~100 lux)
■ Maintenance Wakefulness Test
AN: Adaptation night (8 h); BL: Baseline sleep (8 h) ;
E1 to E9: Extended sleep session (Time in bed = 12 h);
SD: Total sleep deprivation (39 h prolonged wakefulness);
RS: Recovery sleep (12 h)

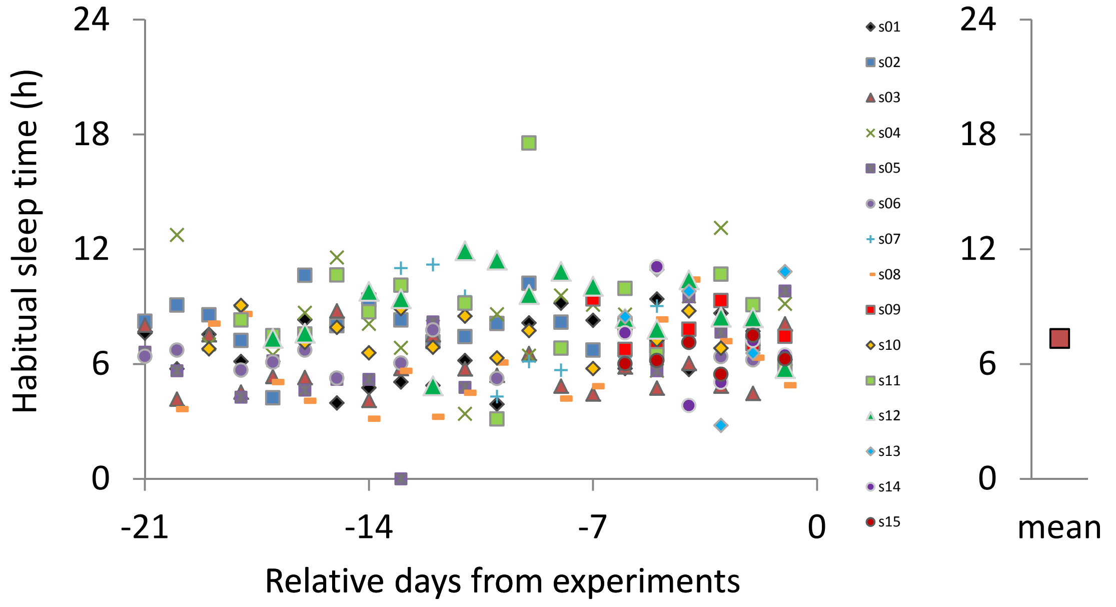

Figure S2 Mean and individual habitual sleep time (N=15)

Left panel: Each symbol represents habitual sleep time in each subject (S01 to S15) over at-home recording. There is large daily variation in each individual (mean range in HST of 6.36±0.82 h). Right panel: Mean of habitual sleep time in all 15 subjects (7.37±0.27 h).

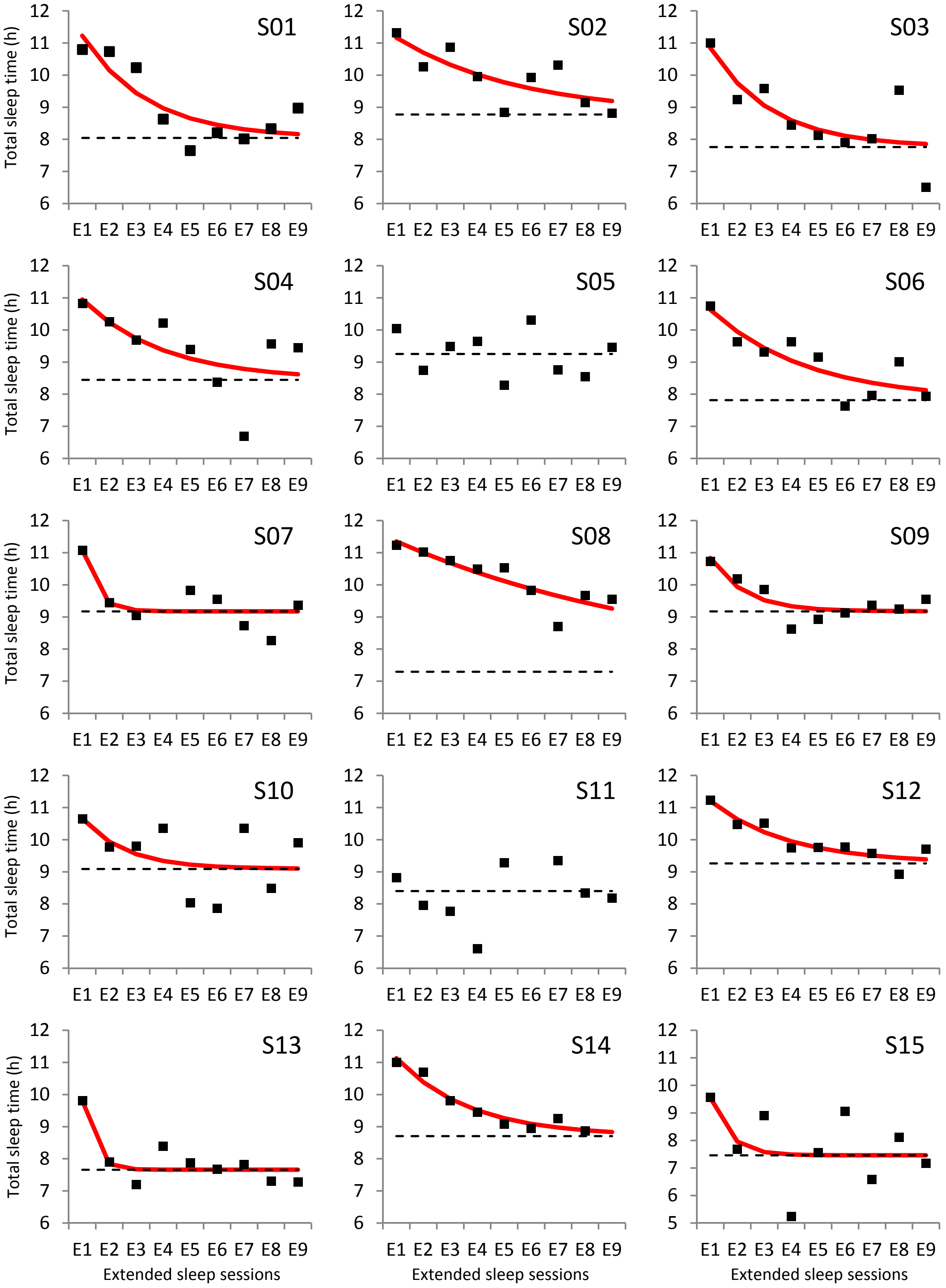

Figure S3 Individual total sleep time in each experimental nights

Each panel contains total sleep time in each subject (S01 to S15) over extended sleep sessions. E1 to E9: Extended sleep session (Time in bed = 12 h); Red solid lines represent fitted exponential decay curve; black hatched lines represent asymptote (the estimated optimal sleep time (OST)). Because not converging for curve fitting, mean TST value over the extended sleep sessions S5 and S11 were treated as the estimate of OST.

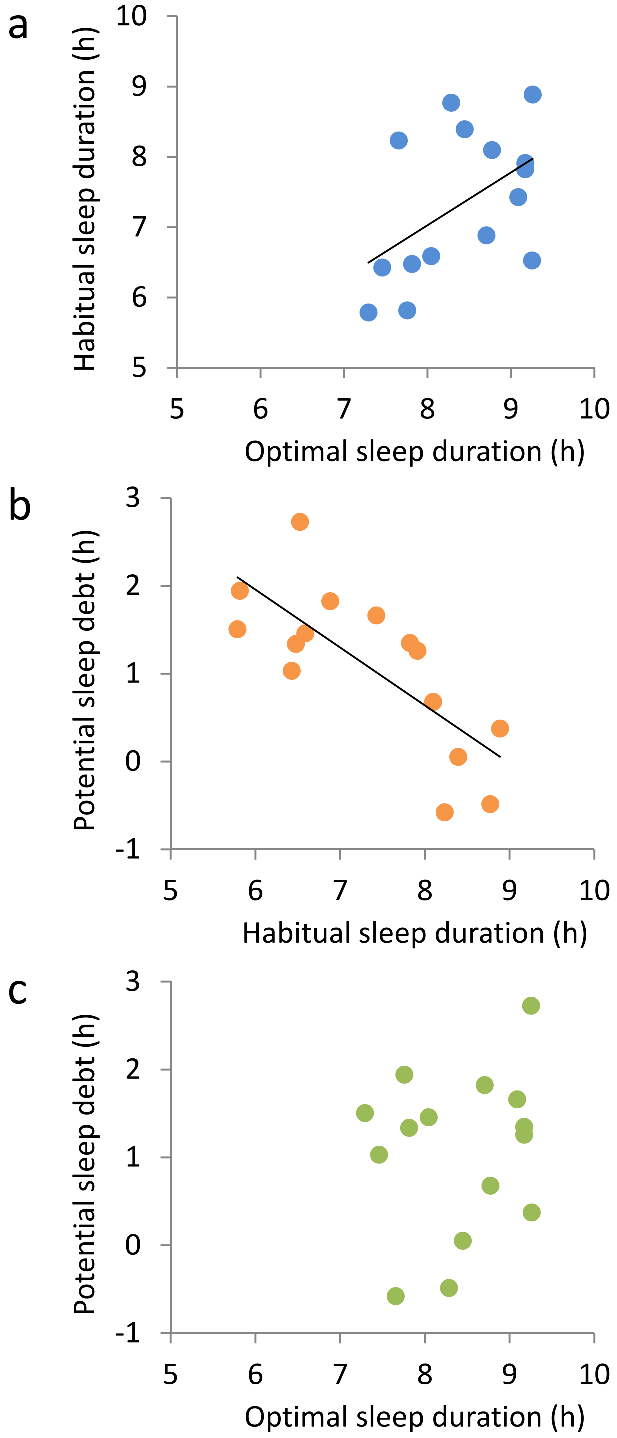

Figure S4

Association between optimal sleep duration (OSD), habitual sleep duration (HSD) and potential sleep debt (PSD)

(a) Although there was a significant correlation between estimated OSD and HSD (r=0.514, p=0.050) HSD was significantly shorter than OSD.
(b) We observed a relationship where PSD increased as HSD became shorter (r=-0.756, p=0.001).
(c) There was no significant correlation between PSD and OSD (r=0.172, p=0.539).

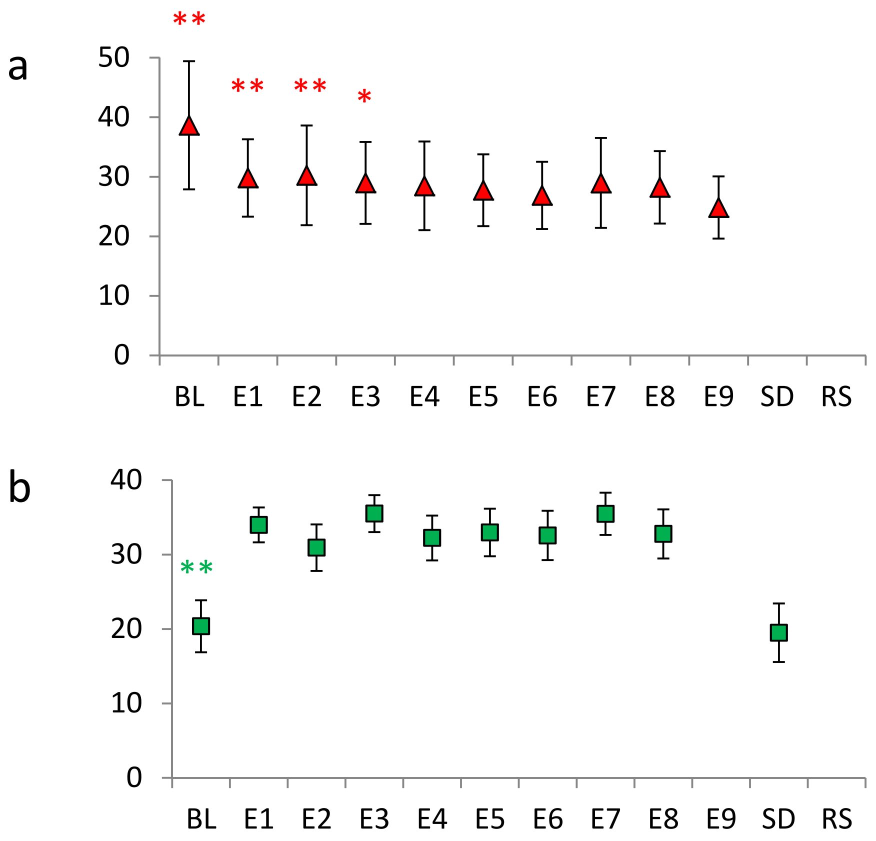

Figure S5 Subjective and objective sleepiness changes

a: Subjective sleepiness (Visual analogue scale) In the wake period. Higher score means more sleepy.
b: Sleep onset latency in the Maintenance Wakefulness Test. Shorter sleep latency indicates stronger tendency for falling asleep.

BL: Baseline sleep (8 h) ; E1 to E9: Extended sleep session (Time in bed = 12 h);
SD: Total sleep deprivation (39 h prolonged wakefulness); RS: Recovery sleep (12 h)

All data are shown as MEAN±SEM. **: $p<0.01$, *; $p<0.05$.(Comparated to E9).

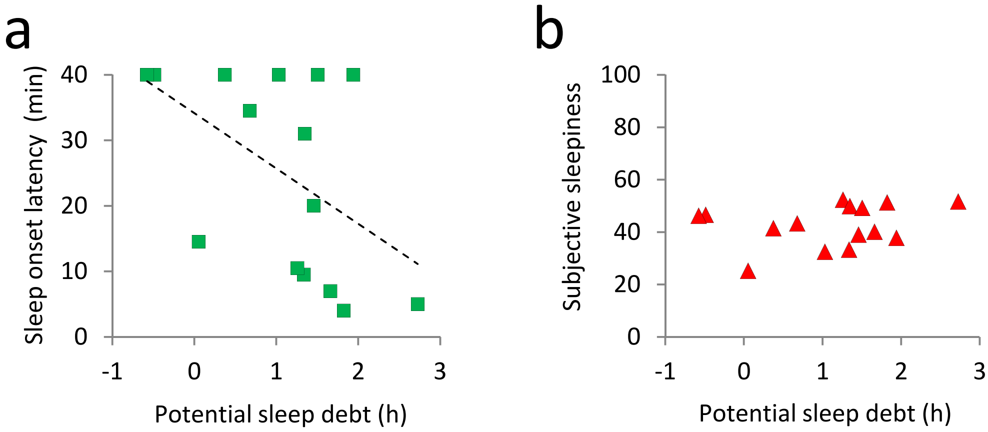

Figure S6 Correlations between potential sleep debt and sleepiness parameters.

Association between Potential sleep debt (PSD) and (a) Maintenance Wakeful Test at B1 and (b) visual analogue scale at B1. There was a significant trend of correlation between PSD and Maintenance Wakefulness Test (r=-0.486, p=0.066) but no correlation between PSD and visual analogue scale (r=0.052, p=0.854)
